# Peritoneal Carcinomatosis Targeting with Tumor Homing Peptides

**DOI:** 10.3390/molecules23051190

**Published:** 2018-05-16

**Authors:** Lorena Simón-Gracia, Hedi Hunt, Tambet Teesalu

**Affiliations:** 1 Laboratory of Cancer Biology, Institute of Biomedicine, Centre of Excellence for Translational Medicine, University of Tartu, Ravila 14b, Tartu 50411, Estonia; lorena.simon.gracia@ut.ee (L.S.-G.); hunt.hedi@gmail.com (H.H.); 2 Cancer Research Center, Sanford-Burnham-Prebys Medical Discovery Institute, 10901 North Torrey Pines Road, La Jolla, CA 92037, USA; 3 Center for Nanomedicine and Department of Cell, Molecular and Developmental Biology, University of California, Santa Barbara, CA 93106, USA

**Keywords:** homing peptide, peritoneal carcinomatosis, intraperitoneal chemotherapy, p32, neuropilin-1, integrins, hyaluronan

## Abstract

Over recent decades multiple therapeutic approaches have been explored for improved management of peritoneally disseminated malignancies—a grim condition known as peritoneal carcinomatosis (PC). Intraperitoneal (IP) administration can be used to achieve elevated local concentration and extended half-life of the drugs in the peritoneal cavity to improve their anticancer efficacy. However, IP-administered chemotherapeutics have a short residence time in the IP space, and are not tumor selective. An increasing body of work suggests that functionalization of drugs and nanoparticles with targeting peptides increases their peritoneal retention and provides a robust and specific tumor binding and penetration that translates into improved therapeutic response. Here we review the progress in affinity targeting of intraperitoneal anticancer compounds, imaging agents and nanoparticles with tumor-homing peptides. We review classes of tumor-homing peptides relevant for PC targeting, payloads for peptide-guided precision delivery, applications for targeted compounds, and the effects of nanoformulation of drugs and imaging agents on affinity-based tumor delivery.

## 1. Introduction

### Challenges in IP Chemotherapy

Primary malignancies of organs of the peritoneal cavity may lead to invasion of malignant cells through visceral serosa, shedding of cancer cells into the peritoneal cavity and their seeding, followed by cellular proliferation and neovascularization of the malignant nodules. Such peritoneal dissemination of malignant disease, peritoneal carcinomatosis (PC), is associated with disease progression and poor prognosis [1,2]. Unfortunately, the PC does not have clear symptoms and is often detected during a late stage of the disease when tumor nodules are widespread over visceral and parietal peritoneum. At this stage, the approved clinical interventions (surgery and systemic chemotherapy) have only a modest effect on the overall survival and the PC is commonly regarded as a terminal condition. In case of colorectal cancer, ~10% of the patients show peritoneal spread at the time of diagnosis [3] and the PC is present in ~40% of terminal patients [4]. In gastric cancer, 20% of patients present PC at the time of surgical resection, and ~60% have PC at death [5]. Finally, the PC mortality in ovarian cancer is ~35% [6]. Thus, there is an urgent unmet need to develop therapies that allow more efficient treatment of peritoneally spread malignancies.

During recent decades a number of therapeutic approaches have been explored for improved management of PC [7]. The PC is a loco-regional disease that is increasingly treated using a multimodal approach—a combination of aggressive cytoreductive surgery, intraperitoneal (IP) chemotherapy and systemic chemotherapy to improve local control of the disease and patient survival. Increased concentrations of anticancer drugs are expected to reach PC lesions after IP administration compared to intravenous administration [8]. IP chemotherapy, administered typically immediately or 2–3 weeks after a cytoreductive debulking surgery, results in elevated local concentration and extended half-life of the drugs in the peritoneal cavity and improves their anticancer efficacy [9,10,11]. Mild heating of the IP-administered drug, a procedure known as Hyperthermic IntraPEritoneal Chemotherapy (HIPEC), improves drug absorption and drug effect with a low exposure to the rest of the body [12]. During the HIPEC procedure, the surgical team will continuously circulate a 42 °C to 43 °C sterile solution containing a chemotherapeutic agent throughout the peritoneal cavity, for a maximum of two h in an attempt to kill remaining cancer cells. A latest addition to IP targeting of cancer drugs is a procedure known as Pressurized IntraPEritoneal Aerosol Chemotherapy (PIPAC) that involves nebulization of drug solution into CO_2_ pneumoperitoneum during laparoscopy [13]. However, to date the IP peritoneal cancer therapies have not become standard of care. IP-administered chemotherapeutics have a short residence time in the IP space, as molecules below 20 kDa are rapidly cleared from the IP cavity via the direct absorption [14] and compounds above 20 kDa and nanoparticles are eliminated by lymphatic drainage through stomata located between cuboidal mesothelial cells of the diaphragm [15]. Short IP retention time necessitates frequent dosing and may lead to later catheterization-related problems such as obstruction, infection, and bowel complication [16]. In addition, the IP chemotherapy is administered using complex and expensive perfusion devices not available in many hospitals. 

Formulation has a profound effect on the pharmacokinetics, biodistribution, and efficacy of the drugs. As summarized in a recent review by Dakwar et al., nano- and microformulation of anticancer compounds can increase peritoneal retention of drugs that translates into an improved therapeutic efficacy and reduction of the number of IP administrations [17]. As nanoparticles in most cases enter cells via endocytosis, loading of drugs in nanoparticles can also bypass or alleviate drug resistance due to overexpression of drug efflux pumps [18,19,20]. In precision cancer medicine, affinity targeting with tumor specific ligands is an increasingly popular approach for improving target selectivity of drugs and decreasing side effects [21]. In particular, a number of systemic tumor selective antibody-drug conjugates (ADC) have been approved or are in clinical trials. In a recent review by Lambert et al. [22], advances in the development of ADC for the treatment of solid tumors are discussed, and the ADCs on market or in clinical trials are summarized (Table 1 in [22]). In addition to the systemic administration route, other administration routes such as local (intratumoral) or loco-regional (e.g., intraperitoneal) routes are explored for precision delivery of payloads [23].

**Figure 1:**
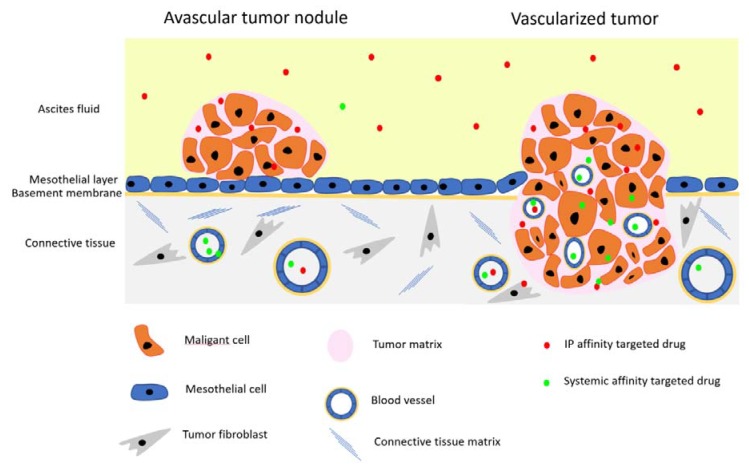
Intraperitoneal (IP) versus systemic targeting of peritoneal carcinomatosis (PC). IP-administered affinity targeted drugs are recruited and penetrate tumors directly and through systemic circulation, whereas intravenous (IV) drugs rely on systemic transport. IP delivery route has the advantage of targeting small avascular tumor nodules left behind after surgery.

In recent years, a number of studies on PC models have explored the effect of affinity targeting on the pharmacokinetics, tissue distribution, and antitumor efficacy of IP-administered cancer therapeutics, nanomedicines in particular. Intraperitoneal affinity targeting can in an ideal case be used to maximize tumor targeting by a dual mechanism: via the direct receptor mediated intraperitoneal uptake to target avascular tumor nodules attached to the peritoneal mesothelial layer and following peritoneal escape via the systemic route to improve targeting of angiogenic vessels in vascularized tumors (Figure 1). Here we review recent progress in active targeting of intraperitoneal anticancer compounds, focusing on the use of small peptides (˂10 aminoacids) to increase the specific tumor binding and penetration of the drugs and imaging agents for PC treatment and imaging of peritoneal tumors.

## 2. Affinity Targeting of Tumors with Homing Peptides

Compared to other tumor-targeting ligands (e.g., antibodies, polysaccharides, aptamers), homing peptides offer several advantages, including small size (typically < 10 aminoacids), affordable manufacturing cost, low immunogenicity, biocompatibility, and moderate affinity that circumvents the affinity site barrier [24,25,26]. Homing peptides as targeting elements are particularly relevant in the context of nanomaterials, where even small molecule targeting ligands with weak affinity can, through multivalent interactions, significantly enhance target-specific avidity (by up to 4 orders of magnitude), and thus the affinity of the final material is readily tunable [27]. Small peptides typically target functionally important binding pockets on target molecules that are highly conserved between the species. This circumstance renders many homing peptides bioactive as receptor agonists or antagonists, and further increases their translational relevance. Compared to more complex ligands (e.g., antibodies) the recent advances in peptide synthesis strategies allow cost-effective synthesis and scale-up of the production [25]. Moreover, chemical modification and/or use of unnatural amino acids can be used to render peptides less susceptible to enzymatic degradation and to improve overall peptide stability, alter structure for improved interaction and biological function, and/or to modulate immunogenicity [25,26,28].

A combinatorial screening method, peptide phage display, has been widely used as an unbiased tool to map molecular landscape of the cell surface and to develop systemic peptidic homing ligands for tissues or diseases of interest [29]. For phage display, random or constrained peptides are expressed on the surface of the recombinant phage nanoparticles as fusions to the phage coat protein, to yield libraries with a typical diversity of ~1 billion–enough for in-depth coverage of all amino acid combinations in a 7 amino acid peptide library. During biopanning, the phages that display binding peptides are retained at the target; corresponding peptides are then chemically synthesized for targeted delivery studies and for peptide binding partner (receptor) identification [30]. Over the years, a number of tumor-homing peptides of different specificities have been identified using in vivo phage display with systemically administered peptide phage libraries [31,32]. Many tumor-homing peptides interact with binding partners overexpressed on the endothelial cells of tumor blood or lymphatic vessels. Another group of tumor-homing peptides binds to target molecules expressed on the surface of the extravascular cells in tumors: to malignant cells (including tumor stem cell like cells), to macrophages and other immune cells, to fibroblasts, or to tumor-associated extracellular matrix isoforms. As intraperitoneal targeting primarily relies on direct (not vascularly mediated) tumor recruitment, this second group of homing peptides is of particular relevance. In addition, our laboratory and others have applied IP in vivo phage display to identify peptides capable for targeting PC lesions in mice [33,34].

Below we describe the reported studies on tumor-homing peptide-based IP targeting of peritoneal tumors. Table 1 summarizes the published peptides used for preclinical targeting of peritoneal lesions, peptide receptor(s), tumor models used in each study and outcomes of the studies.

### 2.1. Integrin Targeting Peptides 

Integrins are a family of 24 structurally related cell surface heterodimers with a role in the interaction of cells with the extracellular matrix and in the cell-cell adhesion [35]—functions that are crucial for the tumor initiation, progression, and metastasis [36]. In particular, the RGD tripeptide motif, recognized by a subset of integrins [37] has been widely used to guide therapeutics, imaging agents, macromolecules, and nanoparticle to tumor vasculature and -parenchyma [38]. RGD-containing peptides have been successfully used for detection and experimental treatment of PC modeled in mice, in particular the αvβ_3_ integrins are relevant in the context of PC as they are highly expressed in peritoneal tumor lesions of gastric- [39], colon- [40], and ovarian carcinoma [41].

In a 2007 study, Dijkgraaf et al. reported homing and antitumor activity of the IP-injected integrin-targeted radiolabeled compounds in OVCAR-3 human ovarian carcinoma model of PC [42]. Cyclic RGD peptide (cRGDfK) conjugated to a complexing agent 1,4,7,10-tetraazacyclododecane-1,4,7,10-tetraacetic acid (DOTA, tetraxetan) and radiolabeled with ^111^In or ^177^Lu was found to preferentially accumulate in the malignant tumor lesions, with ~39% of injected dose (ID)/g found in peritoneal tumors after 4 h of IP injection. In the experimental therapy study, the survival in the group of mice treated with ^177^Lu-DOTA-cRGDfK was significantly increased compared to the untreated control group (21 weeks vs. 5 weeks). This study suggested that IP-administered radiolabeled DOTA-E-c(RGDfK) is suitable for PC targeting and with potential clinical applications in precision radionuclide therapy. 

Cytoreductive surgery is a critical component in the treatment of patients with PC and completeness of cytoreduction has a profound effect on the long-term survival. It is, therefore, of high translational relevance to develop technologies that help achieving precise intraoperative visualization of malignant tumor tissue to allow more complete elimination of tumor tissue, minimize the damage to normal tissues, and reduce time of surgery. A series of studies has addressed the development and preclinical validation of cRGDfK-guided near infrared (NIR) fluorescent dyes. A 2006 study from Dr. Coll’s laboratory described development of a molecular imaging tool composed of four copies of cRGDfK peptide attached to one Cy5 infrared dye (4xcRGDfK/Cy5) for a non-invasive imaging of peritoneally disseminated IGROV1 ovarian carcinoma model in mice [43]. After intravenous injection, >1 mm intra-abdominal malignant nodules became visible in the case of 4xcRGDfK/Cy5, whereas no signal was observed in the mice injected with the monovalent cRGDfK/Cy5, or Cy5 conjugated to four copies of a negative control peptide. The improved targeting of multivalent 4xcRGDfK/Cy5 complex is likely due to a cooperative binding and steric stabilization of the ligand-receptor interaction. In a follow up study, four copies of cRGDfK were coupled to Alexa Fluor700 and used for image-guided surgery [44]. The NIR light-guided surgery allowed detection and removal of nearly twice the amount of tumor nodules compared to resection under white light and the duration of the surgery was reduced from 20 min to 14 min. In another recent study, an imaging probe composed of two copies of RGD peptide coupled to an indocyanine green dye was intravenously injected for NIR imaging-guided resection of PC of gastric origin in mice [24,45]. Remarkably, the application of the RGD-guided system allowed achieving a diagnostic accuracy rate of 94% and sensitivity of 100%. In contrast, the diagnostic accuracy achieved with the conventional surgery was 76%. The guided surgery allowed detection of tumors nodules >1.8 mm in diameter and shortening of the duration of the surgery 3 times compared with the conventionally surgery. The authors concluded that the benefits of their surgical navigation system warrant future clinical development.

Finally, the study by Akita et al. used in vivo IP peptide phage display on PC models of gastric origin to identify SWKLPPS peptide that contains the KLP motif with homology to laminin 5, a ligand of α_3_β_1_ integrin peptide [34]. After IP injection, the synthetic SWKLPPS peptide showed > 60-fold higher binding to peritoneal tumors than the control peptide, and SWKLPPS-guided liposomes also showed significantly higher tumor accumulation than the untargeted liposomes (1.7 vs. 0.85% of injected dose/100 mg wet tissue).

Importantly, peptidic integrin ligands may have intrinsic antitumor activities in the PC. Interactions of integrins on surface of malignant cells with the components of the extracellular matrix play important roles in tumor maintenance, progression and peritoneal dissemination [46]. RGD peptides and peptidomimetics interfere with the tumor cell binding to ECM and affect cellular migration, growth, differentiation, and apoptosis [47,48,49,50]. In one study, the effect of the RGD and YIGSR peptides on the invasiveness of gastric cancer was studied. The survival of mice with gastric cancer treated with YIGSR, RGD, or a polymer containing several copies of RGD was significantly increased [51]. The interpretation was that the peptides block the binding of integrins expressed in the tumor cells to the ECM to inhibit peritoneal dissemination. In another study, the inhibition of peritoneal metastasis in mice with gastric tumor, after treatment with RGD peptide, the pseudo RGD peptide FC-336, or anti-integrin antibody was examined [52]. The peptides or antibody were administered IP after CO_2_ pneumoperitoneum and the frequency and weight of the port-side metastasis was measured. RGD peptide and FC-336 significantly inhibited the frequency and the weight of the port-site metastasis and this inhibition was dose-dependent. 

### 2.2. Tumor-Penetrating Peptides

A subclass of tumor-homing peptides—tumor-penetrating peptides—can be used to increase extravasation and parenchymal accumulation of drugs, imaging agents and nanoparticles in tumors [32]. Tumor-penetrating peptides are defined by the presence of the C-end rule (CendR) motif with the consensus R/KXXR/K (R-Arg, K-Lys, and X-any amino acid) [53,54]. This position-dependent motif must be C-terminally proteolytically unmasked to allow binding to cell and tissue penetration receptor neuropilin-1 (NRP-1) [54,55]. NPR-1 is overexpressed in the tumor vasculature and in variety of tumor cells, including peritoneal tumor cells, in vitro and in vivo [39,40,56]. The binding of the CendR peptide to NRP-1 activates a macropinocytosis-related transport pathway that is regulated by nutrient availability mediates a transcytosis cascade and results in the distribution of the payloads deep into the tumor parenchyma [57]. 

**Figure 2:**
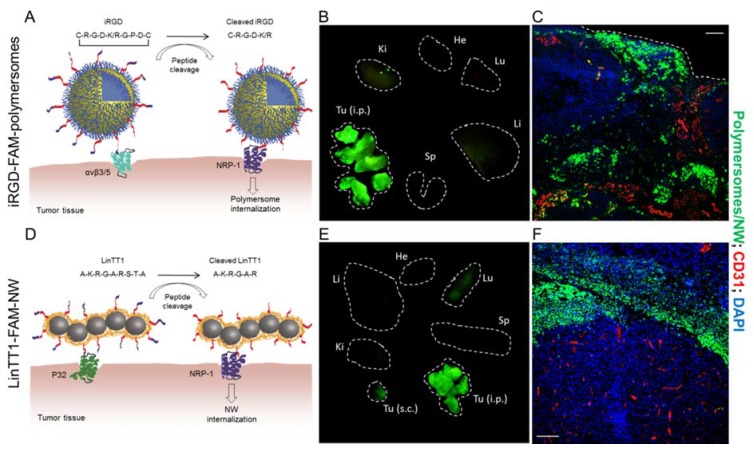
Intraperitoneal (IP) tumor homing and penetration of tumor penetrating CendR (C-end Rule) peptide-targeted nanosystems. (**A**) Schematic representation of fluorescein labeled iRGD-targeting polymersomes (iRGD-FAM-polymersomes) and mechanism of tumor targeting and penetration. pH-sensitive polymersomes made of the copolymer poly(oligoethylene glycol methacrylate)-poly(2-(diisopropylamino)ethyl methacrylate) (POEGMA-PDPA) were functionalized with the iRGD CendR peptide. iRGD on polymersome surface binds to αvβ_3/5_ integrins in the tumor blood vessels via the RGD motive. Upon cell surface recruitment, iRGD is proteolytically processed to expose C-terminally the CendR motif (CRGDK/R) and allow binding to NRP-1 in the tumor tissue. The NRP-1 binding triggers the penetration of the peptide together with the drug-loaded polymersomes into the cells and through tumor tissue. (**B**) PC homing of iRGD-FAM-polymersomes. MKN45-P bearing mouse was injected IP with 20 mg polymer/Kg, perfused 24 h later, and excised organs were subjected to fluorescence imaging ex vivo. (**C**) Confocal microscopy of the peritoneal MKN45-P tumor tissue collected 24 h after IP injection of iRGD-FAM-polymersomes. Green = polymersomes; red = CD21; blue = DAPI. Scale bar: 100 µm. (**D**) Schematic representation of fluorescein-labeled LinTT1-targeted iron oxide nanoworms (LinTT1-FAM-NW). Dextran-coated iron oxide nanoworms (NWs) were functionalized with the CendR peptide LinTT1. LinTT1 on the nanoworms is recruited to p32 receptor on the surface of tumor cells. LinTT1 is cleaved by the tumor related enzyme urokinase plasminogen activator (uPA), exposing the CendR motif (AKRGAR) that binds to neuropilin-1 (NRP-1) and triggers the internalization of the peptide and the attached NW into tumor tissue. (**E**) Ex vivo images showing the specific homing of LinTT1-FAM-NW. MKN45-P bearing mouse was injected IP with 5 mg iron/Kg and perfused 5 h later. (**F**) Confocal microscopy images of the peritoneal MKN45-P tumor tissue 5 h after IP injection of LinTT1-FAM-NW. Green = NWs; red = CD21; blue = DAPI.

The first tumor-penetrating peptide, iRGD (internalizing RGD, sequence: CRGDKGPDC), was identified by in vivo phage display on metastatic xenograft models of prostate cancer in mice [58]. Systemic iRGD homes to and penetrates the tumor tissue using a multistep mechanism (Figure 2A): (1) iRGD is recruited to tumor vessels by interaction of its RGD motif with αvβ_3_ and αvβ_5_ integrins on tumor cells and tumor endothelial cells; (2) The peptide is then proteolytically processed by a tumor-associated protease to C-terminally expose the active CendR motif, CRGDK; (3) the CRGDK fragment of iRGD then binds to NRP-1 to trigger transcytosis cascade that leads to deep penetration of peptide into the tumor tissue (Figure 2A–C). Importantly, for tumor penetration the cargo does not need to be conjugated to iRGD: the peptide increases the accumulation and penetration of drugs and macromolecules co-administered with iRGD—a phenomenon known as a bystander effect [59]. 

Overexpression of iRGD receptors (integrins and NRP-1) in malignant tissues and the ability of iRGD to directly penetrate tumors using non-vascular route [58,59], make iRGD well suited for IP precision delivery. In the MKN-45P gastric PC xenograft model, IP-administered iRGD potentiated the tumor penetration and the anticancer activity of coadministered free doxorubicin [39]. iRGD coadministration potentiated tumor accumulation of doxorubicin ~2.5 fold, whereas it had no effect in the normal organs. iRGD effect on tumor accumulation and penetration of doxorubicin was independent of circulation, suggesting that small and poorly vascularized peritoneal tumors are directly targeted [50]. 

In a series of recent studies, we have explored potentiating effect of the tumor-penetrating peptide functionalization on PC targeting of nanoparticles. First, we established baseline MKN-45P gastric tumor accumulation for IP-injected non-targeted pH-sensitive polymersomes, a nanoscale platform we chose as it allows efficient endosomal escape and cytosolic release of payloads [60]. pH-sensitive polymersomes retain the drug at physiological pH and disassemble and rupture endosomes due to proton sponge effect at pH < 6.5. We saw a significant decrease in the growth of peritoneal tumors in mice treated with the paclitaxel-loaded polymersomes compared to mice treated with free drug and with paclitaxel-albumin nanoparticles (Abraxane^®^, Summit, NJ, USA). In a follow-up study, we studied whether tumor homing and anticancer activity of polymersomes can be further potentiated by iRGD functionalization [40]. We observed that gastric PC homing and penetration of the IP-dosed Paclitaxel-loaded polymersomes was increased by coating the polymersomes with iRGD peptide (Figure 2A–C). This enhanced tumor accumulation translated into potentiated antitumor efficacy and reduction of the number of peritoneally disseminated tumor nodules. iRGD-polymersomes accumulated in peritoneal tumors by combination of direct penetration from the peritoneal space and systemic homing after the escape of the polymersomes into the circulation. Polymersomes are smaller than the size of the lymphatic stomata in the peritoneal mesothelial layer (500 nm) and thus expected to be cleared from IP space via this route into the systemic circulation. Such dual homing is of great interest as it allows simultaneous targeting of both small poorly vascularized peritoneal tumor nodules (via direct peritoneal targeting) and larger highly vascularized tumor lesions (via systemic homing). 

Another nanoplatform that holds a great promise for targeted tumor delivery, especially for nucleic acid payloads, is peptide guided cationic liposome nanoparticles [61]. We studied biodistribution and peritoneal tumor penetration of cRGD and iRGD-targeted polyethylene glycol (PEG)-stabilized cationic liposomes in mice bearing peritoneal carcinomatosis derived from human MKN-45P gastric tumor [61]. Whereas the IP-dosed untargeted cationic liposomes showed some charge-mediated tumor accumulation, the tumor homing and penetration were increased by functionalization with iRGD or cRGD peptides. Importantly, the affinity-targeted liposomes preferentially penetrated small (<0.3 mm) peritoneal tumor nodules—a clinically highly relevant target that is left behind during the cytoreductive surgery and drives the peritoneal dissemination and tumor recurrence. 

In recent years, several other tumor-penetrating peptides have been identified and validated for precision tumor delivery [27,32]. Cyclic CKRGARSTC peptide (codenamed “TT1”) was identified by phage biopanning on p32 purified protein [62] and validated as an affinity ligand in different tumor models and administration routes, including in PC models and IP administration [63,64,65]. Both cyclic TT1 and its linear lower-affinity variant, linear TT1 (“LinTT1”, sequence: AKRGARSTA), bind to p32 protein (also known as gC1qR or hyaluronic acid binding protein 1, HABP1), an intracellular protein aberrantly expressed on the surface of activated tumor cells, vascular/lymphatic endothelial cells, and macrophages/myeloid cells in hypoxic areas of the tumor [66]. Another p32-ligand peptide identified by phage display, Lyp-1 (sequence: CGNKRTRGC) [66,67,68]. showed intrinsic antitumor activity in a breast tumor model in mice [69], and accumulation in p32-expressing artherosclerotic plaques in mice [69]. After p32 binding, cryptic CendR motif (KRGAR) in TT1 and LinTT1 peptides is proteolytically exposed by the tumor-associated protease urokinase type plasminogen activator (uPA), to trigger NRP-1 binding and promote the tumor penetration [63] (Figure 2D). We have recently shown that, upon IP administration, the paramagnetic iron oxide nanoworms (NW) targeted with LinTT1 (LinTT1-NW) specifically home to and penetrated the peritoneal tumors of gastric, colon, and ovarian origin in mice (Figure 2D–F) [64]. NWs targeted with the LinTT1 peptide allowed tumor imaging using T2 magnetic resonance imaging. IP treatment of PC mouse models with NWs coated with LinTT1 in tandem with a pro-apoptotic peptide resulted in significant reduction of the peritoneal tumor growth and the number of tumor nodules compared with the untargeted NWs. As LinTT1-NWs were also found to promote tumor penetration of a co-administered cargo (fluorescently labeled 70 kDa dextran) the peptide may have applications for combination delivery of free coadministered anticancer drugs and/or imaging agents to the PC lesions. 

### 2.3. M2 Macrophage-Targeting Peptide

Tumor-associated M2-skewed macrophages (M2 TAMs) are increasingly recognized as important players in the tumor progression and maintenance. M2 TAMs contribute to immunosuppressive environment in tumors, promote tumor angiogenesis and metastasis [70,71] and contribute to tumor relapse after the chemotherapy [72]. In the context of peritoneal carcinomatosis, IP M2 TAMs are known to contribute to the progression and spreading of gastric cancer with peritoneal dissemination [73]. We have recently identified a 9-residue cyclic peptide CSPGAKVRC (“UNO”) that selectively targets M2 TAMs in vivo [74]. UNO peptide was identified by in vivo phage display by IP injection of the phage library into mice bearing 4T1 breast tumors. In mechanistic homing studies, we found that UNO acquires the ability to interact with its cellular binding partner CD206 (also known as mannose receptor, a cell-surface receptor upregulated in M2 TAMs, only after the disulfide bridge in the peptide becomes reduced. As many solid tumors are in a state of imbalance favoring a reducing environment and reductive stress [75], UNO binding is restricted to CD206-expressing TAMs in the tumor tissue. Systemically administered UNO peptide accumulated inside CD206-expressing TAMs across a spectrum of solid tumors, including peritoneally disseminated gastric tumors. Specific accumulation of UNO in M2 TAMs in peritoneal tumors suggests that its cytotoxic drug conjugates may have applications in therapeutic depletion of M2 TAMs to suppress peritoneal cancer dissemination.

### 2.4. Nucleolin Targeting Peptide

The F3 peptide is a 31-residue peptide discovered by biopanning of cDNA phage library on cultured progenitor cell-enriched bone marrow cells and in vivo on HL-60 human leukemia cell tumor xenograft [76]. The F3 sequence corresponds to N-terminal fragment of human high mobility group protein 2 (HMGN2), a protein known to be associated with chromatin in a cell cycle-dependent manner [77]. The F3 peptide targets nucleolin [78], a protein expressed in the nucleus of resting cells, but cycling between the cell nucleus and the plasma membrane in activated cells, including in malignant tumor cells [79]. The cell surface expression of nucleolin in proliferating cells and its intracellular shuttling render the F3 peptide an attractive targeting moiety for intracellular cargo delivery. 

To explore the suitability of F3 for precision targeting of PC, mice bearing IP MDA-MB-435S tumors were subjected to an experimental IP therapy with dimeric F3 peptide coupled to the alpha-emitter ^213^Bi [80]. Remarkably, following IP injection, ~32% of the ID/g was found to accumulate in the tumor tissue. In contrast, no significant accumulation of the F3-targeted alpha-emitter was detected in control organs, except for the kidneys due to the renal excretion. Experimental therapy with the IP-administered F3-targeted alpha-emitter showed a significant increase of survival (average survival of 93 days) compared with the non-targeted alpha-therapy (average survival of 53 days). In addition, the number of peritoneal tumor nodules was lower in the animals subjected to F3-guided alpha-therapy. Another study compared two different F3-guided alpha-emitters, ^213^Bi and ^225^Ac, and found more pronounced decrease of the number of peritoneal tumors nodules after treatment with ^213^Bi radioisotope, most probably due to its shorter half-life [81]. Importantly and of translational relevance, the antitumor activity of F3-guided ^213^Bi is most pronounced against small tumor nodules that, when left untreated, give rise to disseminated peritoneal tumors. In a follow up study, the group of Dr. Essler performed a combined experimental therapy of mice bearing intraperitoneal OVCAR-3 xenograft tumors with intraperitoneal F3-targeted ^213^Bi and Paclitaxel [82]. The mean survival of the mice that received IP combined therapy was significantly longer than of the mice treated with both therapeutic agents separately (121 days vs. 84 days for F3-targeted ^213^Bi and 40 days for Paclitaxel). These studies suggest that multimodal therapy that combines F3 peptide-guided radionuclide therapy, both alone and in combination with cytotoxic drugs, is a promising therapeutic concept. 

### 2.5. EphA2 Targeting Peptide

The EphA2 receptor (ephrin type-A receptor 2) is a receptor tyrosine kinase with roles in the regulation of tumor cell growth, migration, invasion, and angiogenesis upregulated in a variety of solid tumors, including IP ovarian cancer [83,84]. Phage biopanning on EphA2 was used to identify ephrin-mimicking peptide YSAYPDSVPMMS (YSA) with submicromolar affinity towards the receptor [85]. In a study, the IP YSA-functionalized magnetic cobalt spinel ferrite (CoFe_2_O_4_) nanoparticles were used to selectively extract metastasizing ovarian cancer cells from peritoneal effusions [86]. The YSA magnetic nanoparticles were injected IP in mice injected with fluorescently labeled Hey ovarian carcinoma cells known to be strongly positive for of EphA2 receptor expression). After magnetic field exposure, the fluorescent Hey cells were captured together with the targeted magnetic nanoparticles in the peritoneal cavity. To test the specificity of the cellular capture, the YSA-nanoparticles were IP injected in a mouse injected with both Hey cells and BG-1 cells (the latter with a low peptide receptor expression). After cell extraction from the peritoneal cavity and magnetic separation, more than the 95% of recovered cells were Hey cells. In a follow up study, the YSA-magnetic nanoparticle platform showed promise in capture of ovarian cancer cells dissociated in human ascites fluid in vitro [87]. 

These studies on peptide-mediated extraction of cell lines are translationally important, as the strategy can be used to eliminate exfoliating cancer cells and malignant cells that may escape during primary tumor excision, that are responsible for peritoneal tumor dissemination. In combination with routine treatment procedures selective extraction of free cancerous cells in the IP space could be used to improve long-term survival of cancer patients.

### 2.6. Hyaluronan Targeting Peptide 

Hyaluronic acid (HA) is a glycosaminoglycan component of the extracellular matrix present in epithelial, connective, and neural tissues. In peritoneal space, HA is present on mesothelial surface, prominently expressed in peritoneal tumors of gastrointestinal [33]. and ovarian origin, where it is known to contribute to the peritoneal dissemination [88]. We have recently identified a hyaluronan-binding peptide that targets peritoneal tumors [33]. The 9-residue cyclic peptide CKRDLSRRC (IP3) was identified by in vitro and in vivo phage display in a mouse model of PC derived from MKN-45P gastric tumor. After IP injection, the fluorescent-labeled IP3 and silver nanoparticles functionalized with IP3 specifically targeted and penetrated the peritoneal tumors of gastric and colon origin, suggesting that IP3 could also guide therapeutics and imaging agents to peritoneal lesions. Importantly, the peptide is internalized in tumor cells, possibly through receptors such as CD44 and HA receptors for endocytosis (HARE/Stab2).

## 3. Conclusions and Perspectives

The clinical management of peritoneal malignances has evolved over the last thirty years, with the main changes being introduction of cytoreductive surgery in combination with IPC/HIPEC, and more recently, PIPAC. However, IP chemotherapeutics are subject to rapid lymphatic and adsorption-driven clearance, exhibit local toxicity and have limited penetration depth in malignant lesions. For best therapeutic response a PC drug should, upon IP administration, be retained in the peritoneal space and be specifically recruited to the malignant lesions and penetrate the tumors to cause malignant cell death while keeping pan-peritoneal and systemic toxicities low. 

Application of nanoparticles addresses some of the challenges related to IP delivery to peritoneal tumor lesions. A careful optimization of nanocarrier properties—size, shape, charge density, stability in the tumor peritoneal fluid—can maximize tumor uptake via the direct intraperitoneal and systemic routes to improve simultaneous targeting of both avascular tumor nodules and larger tumors. Nanoparticles, especially nanoparticles that allow plasmonic enhancement of fluorophore signals, can also become important as tools for visualization of PC lesions for image-guided surgery. Conditional biocompatible nanoparticles that can be eliminated by exposure to mild etching solution [89,90,91] to allow visualization of only internalized nanoparticles may become of particular interest in the context of PC imaging. 

Functionalization of drugs and nanoparticles with targeting peptides increases their peritoneal retention and provides a robust and specific tumor binding and penetration that translates into improved therapeutic response. Historically, the most widely used strategy for the peptide-mediated affinity targeting of peritoneal tumors has been the targeting of α_v_-integrins with RGD-containing peptides. A new member of the RGD family of tumor-homing peptides, iRGD tumor-penetrating peptide, provides improved tumor penetration that translates into improved efficacy for iRGD-conjugated drugs and nanoparticles. Importantly, iRGD and other tumor-penetrating peptides can deliver coadministered payloads to the tumor, a phenomenon known as bystander effect. An important feature of the combination delivery is that it is not limited by the number of peptide receptors and may thus allow delivery of more therapeutic and imaging payloads than is possible with conjugated delivery systems.

In vivo peptide phage display has expanded the arsenal of peptidic targeting ligands available for precision targeting of the PC. A current trend in PC, already seen during discovery of systemic tumor-homing peptides, is development of tumor-homing peptides that specifically target particular cell populations of interest, such as tumor associated M2-skewed macrophages for UNO peptide, or tumor associated macrophages/lymphatics for p32 targeting peptides (LyP-1 and TT1 family). It is expected that agnostic in vivo peritoneal phage display in combination with isolation of cell populations of interest will be used to further refine the tumor-targeting options for the PC lesions. Immediate target cell populations of therapeutic interest are tumor stem cell-like cells, tumor-associated fibroblasts, and immune cells. 

We envision that an availability-expanded arsenal of tumor-homing peptides and optimized nanocarriers allows multipronged and personally adjusted approaches to PC drug delivery.

## Figures and Tables

**Table 1:** Peptide-targeted systems for treatment, imaging and guided surgery of PC.

Targeting Peptide	Cargo (Drug or Imaging Molecule)	Target	Animal Tumor Model	Application	Outcome	References
**c(RGDfK)**	DOTA (tetraxetan)-^111^In/^177^Lu	αvβ_3/5_	OVCAR-3	Tumor treatment	Significant increase in survival.	[42]
	Cy5 dye	Integrin	IGROV1	Guided tumor resection	Detection of 1-to 5-mm IP tumor nodules.	[43]
	Alexa Fluor700		TSA-pGL3	Guided tumor resection	2-fold increase in sensitivity detection of tumors; surgery time reduced from 20 to 14 min.	[44]
	Indocyanine green		SGC-7901	Guided tumor resection	Detection of 1.8 mm tumors; the operative time was shortened by 3-fold.	[45]
**iRGD** (CRGDKGPDC)	Fluorescein; Doxorubicin	αvβ_3/5_ Integrin/NRP-1	MKN45P; LOVO-6; IGROV-1	PC treatment and imaging	250% more DOX accumulation in tumor; significant tumor growth reduction in MKN45P model.	[39]
	PTX-loaded polymersomes		MKN45P; CT26	PC treatment and imaging	Significant tumor growth reduction in MKN45P model.	[40]
**KLP** (SWKLPPS)	Adriamycin-encapsulated liposomes	α_3_β_1_ Integrin	AZ-P7a		Significantly higher binding to peritoneal tumors compared with control liposomes.	[34]
**LinTT1** (AKRGARSTA)	Apoptotic peptide-iron oxide nanoworms	P32/gC1qR	MKN45P; CT26; SKOV-3	PC treatment and imaging	Significant tumor growth reduction in MKN45P.	[65]
**IP3** (CKRDLSRRC)	Fluorescein; Silver NP	Hyaluronic acid	MKN45P; CT26		Specific IP tumor target and penetration.	[33]
**UNO** (CSPGAKVRC)	Fluorescein; polymersomes	CD206/MRC1	MKN45P		Specific targeting of M2 macrophages in peritoneal tumors.	[74]
**F3** (KDEPQRRSARLSAKPAPPKPEPKPKKAPAKK)	^213^Bi; ^225^Ac	Nucleolin	MDA-MB-435S OVCAR-3	PC treatment	Significant survival increase; decrease of the number of peritoneal tumors.	[81,82,83]
	^213^Bi, combined with PTX			PC treatment	Significant survival increase but not complete remission.	
**YSA** (YSAYPDSVPMMS)	Magnetic nanoparticles	EphA2	Hey	PC treatment	Removal of tumor cells from IP cavity.	[87]
					Removal of ovarian cancer cells from ascites in vitro.	[88]
